# Parklife and the public: 40 years of personalization in the United Kingdom

**DOI:** 10.1080/1600910X.2023.2173267

**Published:** 2023-02-13

**Authors:** William Viney

**Affiliations:** Department of Anthropology, Goldsmiths, University of London, London, UK

**Keywords:** Personalization, history, public services, health, social care, new public management, United Kingdom

## Abstract

This article historicises practices called ‘personalization’ in the UK. It presents data from interviews with practitioners to show how business leaders, public sector managers, policy analysts, activists and others have crafted their personalizing practices through commercial and governmental work over a 40-year period. These public histories are illuminated by professional biographies, which reveal common interests in the transfer and application of technology, data and data analytics. Yet they also illuminate attempts to reform bureaucracies in public and private sectors alike during the late 20th and early twenty-first century. The article asks how mobility – of professionals and their personalizing practices – has affected the pre-existing contrast between, and separation of, public and private organizations. This article contributes to commentaries on personalization that view it as an essentially private and privatizing process. What remains of such domains once they have been crossed, and from whose perspective? This UK history raises questions for comparative histories of personalization and its synonyms elsewhere.

## 1. Introduction: power to the person

This article connects biography and history to explore the political and historical contexts of personalization in the United Kingdom. In 1988, a 29 year-old man called Charles Leadbeater published an essay that analyzed the politics of contemporary individualism. The essay, ‘Power to the Person’, was published by *Marxism Today* – a small yet influential left-wing magazine.^1^ Leadbeater reflected on the Conservative government’s ideological strength, which had brought Margret Thatcher three successive election victories. For Leadbeater these victories were based on her party’s appropriation of individual desire, the corporate capture of values such as autonomy, responsibility, and freedom from the state. ‘Thatcherism’, said Leadbeater, described the reactionary power to convince people that such desires could not be met by state services. The state was viewed by the political right as too uniform, inefficient, infantilising and discriminatory. The problem for Leadbeater was not individualism as such. He felt there was nothing inherently wrong about self-interest but the ‘individual consumerism’ promoted by Thatcher’s government meant market provision alone promised self-sufficiency, choice and increased standards of living. This was worth challenging, thought Leadbeater. There are ways for the state to empower citizens and promote choice without handing over power to markets.

Power to a person (singular) depended on empowering people (plural) – not the one but the many.^2^ Leadbeater proposed a more socially-cohesive and collectivist form of ‘individual citizenship’ or ‘progressive individualism’ (1988, 14). He also asked readers of *Marxism Today* to imagine social life like a park. The state’s role, he said, should be like estate managers. The park would be regulated, allowing a mixed economy with visitors free to choose from a range of services: ‘some the state will provide directly (boats on lakes); many others may involve companies (ice-cream vans) or simply individuals doing what they want with the state's help (sunbathing). The state is vital to ensuring a space continues to exist and is developed; but beyond that *its direct role depends on whether it is the most efficient provider of services*’ (1988, 19, italics added). The metaphor illustrates two things. First, what Leadbeater called progressive individualism would flourish in a system where the state provides some services but regulates those provided by others. According to the terms of the metaphor, all or none of the services could be run by private business, while the state keeps the keys to the park gates. Ultimately, the mix of provision is contingent on performance measures of efficiency, requiring both private and public services to be audited by the same measures of performance. Second, the metaphor and its idiosyncratic details highlight its ideological status. The parkland of ‘progressive individualism’ was an aspirational and promissory vision – born of 9 years of political defeat. In the following 10 years the balance between ideology and practice shifted, new terms would be required.

In 1988 ‘progressive individualism’ was a term Leadbeater gave to a form of a government that managed, oversaw, regulated, facilitated but let private enterprise flourish, without specifying the technical or technological means for doing so. By 1997 and the election of Tony Blair as Prime Minister, Mulgan and Leadbeater entered government as key advisors and directors of policy and strategy units. It is not the purpose of this article to draw a clear line between Leadbeater’s park utopia and the New Labour project that followed. Think tanks and other organizations associated with the Labour party in the early 1990s welcomed post-Fordist, just-in-time production methods and supply-chain management approaches into government (see Mohan 1995; Pautz 2012; Thorpe 2010). They considered the consequences of data-intensive technologies and ‘lean’ production methods to optimize choice in, and responsiveness to, public sector delivery: ‘Retailers have the technology to record thousands of real time changes in people’s preferences … ’ wrote Leadbeater with Demos co-founder, Geoff Mulgan, ‘There is no reason why this technology should not be applied to politics: a just-in-time democracy. The democratic possibilities of combining the television, personal computer and information superhighway have hardly been explored’ (Leadbeater and Mulgan 1994, 61). The activation and political revaluation of ‘people’s preferences’ was stimulated by a wider set of social changes that are represented by these particular individuals.

Think tanks produce expertise by shaping the possibility of local and national policy. As Diane Stone (2000) and Dieter Plehwe (2015) have observed, the integration of think tanks into elite governance has affected how policy is made, by whom, and in whose interests. In the early 2000s Leadbeater published an influential and widely-cited pamphlet, also produced by the think tank Demos, which was co-founded by former *Marxism Today* editor Martin Jacques and fellow contributor, Geoff Mulgan. Leadbeater returned to the utopian terms of ‘Power to the Person’ and the relationship between individual preference and population benefit: ‘The aim of personalised public services is not to provide the self-interested, self-gratification of consumerism’ he wrote, ‘but to build a sense of self-actualisation, self-realisation and self-enhancement’ (2004, 82). The word ‘personalization’ became a term adopted by think tank writers, policy advisors and analysts such as Leadbeater, Mulgan and allied academics, voluntary sector organizations and other groups (see Cribb and Owens 2010; Kelly, Mulgan, and Muers 2002; Leadbeater 2004; Pearson et al. 2020). Though there was no overall consensus about what personalized public services meant for others working in the public sector at the time, for Leadbeater it involved a combination of new managerialism and private innovation, democratic and consumer choice and the application of internet technologies. The term was newly applied – a neologism in the field of public policy – but it had deeper, personal and historical roots related to changes in UK national and international governance, commerce, policy formation, and technological change. The park and its administrative functionaries were dynamic and redistributed, promising an inter-connected space where a person’s choices were met via interpersonal and in silico mediators. These allowed users to access the keys and to be in control of the services they access, and to make decisions about how those services were organized:

Personalisation is . . . simple: by putting users at the heart of services, enabling them to become participants in the design and delivery, services will be more effective by mobilising millions of people as co-producers of the public goods they value, Personalisation has the potential to reorganise the way we create public goods and deliver public services. (Leadbeater 2004, 19)

Replete with the language of adaptation, innovation and disruption common to technology industries (2004, 25), Leadbeater’s ‘personalized’ state is no longer held in a single utopian image nor was the state understood in a purely regulatory or executive role over national parklife. Leadbeater’s personalized state promoted a constantly changing and continuously negotiated reorganization of participant activity for dynamic benefits. The outcomes were then unclear but the organizational consequences were expected to be far reaching. The aspirational language of ‘personalized state services’ rapidly entered mainstream government policy. Shortly before the financial crisis of 2008, the Department of Health predicted that ‘reforming social care to achieve personalization for all will require a huge cultural, transformational and transactional change in all parts of the system, not just in social care but also for services across the whole of local government and the wider public sector’ (2008, 5). Mass personalization was expected to transform all parts of government activity. Every part of local and national government was expected to change. This article will examine the histories between a call for ‘progressive individualism’ and the rise of ‘personalization’ some years later, while exploring the different meanings of personalization, its rapid ascendency into public policy, and some of the different practices that underpin the rhetorical drive towards ‘personalized public services’.

## 2. Methods: putting people into the history of personalization

I began with a brief and narrow history, a history of individuals who moved from a peripheral political position, framed by the ideological struggles of the 1980s and 1990s, to the centre of public sector reform. It is important to stress that ‘personalization’ as a policy term arrived late to this chronology of rhetorical and political transformation. It is a word that interacts with other practices from other social contexts. As this article will show the word remains contested, its history mixed, its practices a patchwork. Even in the narrow UK history related in the Introduction, Leadbeater, Mulgan and others – influencing and directing government policy in the early 2000s – did not act alone. They operated in a political, social, economic and technological environment that facilitated and advanced their ideas. It is this wider environment that this article reports and reflects upon, in order to ask the histories that have shaped personalization in the UK.

Existing academic and practitioner accounts of personalization in the UK public sector tend to focus on the policies managed by particular administrative departments: education (Williamson 2017), policing, security and defense (Amoore 2020; Aradau and Blanke 2022), health and medicine (Feiler et al. 2017; Hedgecoe 2004; Prainsack 2017; Tutton 2012) and social care (Needham 2011; Spicker 2012). Such analyzes follow government policy as a way of tracing wider impacts of ‘personalization’ within specific functions of government. Paradoxically, while academic commentaries focus on personalization as it enters specific government sectors rather than across them, practices associated with personalization are routinely associated with large internet-based companies such as Microsoft, Apple, Google, Facebook and Amazon, that are attributed a vanguard position in a ‘personalization industry’ (Cohen 2019; see also Kant 2020; Prainsack 2017). Accordingly, it is digital media companies, not nation states and their government sectors, that can achieve ‘mass personalization’ (Yeung 2018). The capacity to personalize and target products, services and advertisements, based on algorithmic recommendation systems, is a recent commercial achievement typified by the activities of these online advertising and retail companies (see Lury and Day 2019). For instance, focussing on the technical invention of personalized recommendation algorithms provides a tech-based chronology to personalization, one that emphasizes participative, statistical techniques of tracking, profiling and converting preferences into simplified and scaled data ratings: ‘to follow these individual stories, targeting users not through generic demographic profiles, but with personalized recommendations’ (Seaver 2012). ‘Collaborative filtering’ – just one technique now widespread in personalized approaches to advertising, digital media and retail – involves users rating items with a filter forming suggestions drawn from the ratings of similar users. Collaborative filtering may form recommendations to new collectives of user. Following this logic, which privatizes personalization based on what, and from whom, a personalized intervention is served, contemporary state services can now be evaluated in terms of ‘how much’ the state behaves like internet technology companies. Or how much companies govern on their behalf.

Parallel and related analytic tendencies can conflate ‘personalization’ with the ‘privatiation’ of the state and its services. As this article will show, personalized products and services are historical composites with contested, contemporary statuses. Personalized public services are also embedded in material pasts as they were prospected in techno-political futures. This article aims to supplement existing literatures on personalization in two ways: (1) by examining the historical formation of personalized public services in the UK in terms of professional careers and practices; and (2) by exploring connections and contrasts across different personalizing practices, industries, and areas of service delivery and policy. This article describes what motivated politicians, policy makers, and consultants to change public services, and why the adoption and development of information technologies was one but not the only route towards more ‘personalized’ UK public services.

Our interview participants provided perspectives on personalization where digital technologies and the private sector as such played a greater or lesser role, depending on a person’s historical position. An interview series that asked ‘what is personalization?’ also invited normative statements that combined overlapping and contested histories, their desires and ambitions. To this end, the article as a whole re-examines the implicit or overt confluence made between public sector ‘privatization’, on the one hand, and ‘personalization’ in the development of UK public sector services on the other. It does so by paying attention to why individuals and groups supported and valued and invested in targeting different people.

One additional way that this article contributes to academic literatures about personalization is that it uses summary conclusions from a series of interviews conducted 2018–20 with professionals who identified themselves as practitioners and critics of personalization in the UK. In total, members of our research group carried out 32 semi-structured interviews of approximately 1–1.5 hours in length, across a broad cross-section of professionals from the healthcare, education, social care, marketing and advertising industries. Interviews were conducted in person or remotely. The interviews were recorded and transcribed verbatim. Interviews followed a biographical method used elsewhere in our group’s ethnographic research (see Viney et al. 2022), following a flexible topic guide that began with a person’s biographical background, professional motivations, and careers to date, and emphasized practices that shaped ‘personalized’ goods and services. Interview transcripts were read and discussed with other members of the ‘People Like You’ research group, shared themes identified and further interviews coded.^3^ This article presents a sub-sample of 5 individuals selected from the broader group of interviews because, while they shared common experiences of health and social care as sectors, their careers varied in length, trajectory within and across professional sectors, and varied in terms of their relationship to digital technologies. The interview summaries or ‘portraits’ suggest cases (Forrester 2016) that will be later analyzed in terms of wider changes in policy and public sector management. The selection presented in this article reveals common themes across our interviews: ‘sectors’ are porous and plural, and definitions and practices associated with personalization vary; interviews with professionals of different career trajectories revealed contradictory and context-specific experiences of personalization, that contrast with definitions of personalization that emphasize a particular technology, technique or sector. Each interview gathered a constellation of reported ideas, practices, and employment trends that informed this biographical case analysis of how personalization emerged in different public and quasi-public services. The next section will introduce each person as a ‘portrait’ with a focus on the practices they told us were formative to their views on personalization. There follows a discussion of how the histories of personalizing practices in health and social care are located in longstanding debates about equity, choice, rights, autonomy, economic and technological change. I review the wider changes in the provision and delivery of public services, particularly the impact of New Public Management (NPM). The article concludes by asking whether, and for what purpose, there can be a shared history of personalization.

## 3. Findings: five portraits of personalization

*Every time you implement it, it gets another tweak, improvement, iteratively enhanced kind of model. Our datasets grows. We have more insight, more data to draw on* – **Richard, management consultant**.

Our interview participants reported knowledge and experience of different policy areas. These experiences were tightly entwined with their career trajectories, the acquisition and application of skills in different contexts. Over his career Richard^4^ moved from charitable, commercial, advisory and governmental positions, then returned to the private sector as a management consultant. When I interviewed him, his consultancy was advising local authorities in the UK. Naming and historicizing ‘personalization’ emerged as a common problem during interviews. Reflecting on the relationships between practices – the terms used at different times and in different contexts – many interviewed rejected the term and preferred other words. Tailored, stratified, targeted, segmented, individualized – among other terms. Our interview topic guide explored these cognate terms and the different histories and divergent practices they registered.

Personalization, Richard explained, involved a set of ideas and principles he was familiar with before the word was widely used. His first policy job was for a non-governmental organization (NGO), developing educational and careers programmes. Aligned to the culture of consumer choice Leadbeater was eager to confront, Conservative governments of the 1980s and 1990s sought to stimulate market competition and choice into service areas like careers advice. In this instance, Richard helped to develop a scheme that allowed young people to ‘shop’ for careers advice using a voucher system. In theory, this meant providers could compete in a quasi-market. ‘I guess, probably, if you really wanted to push it, you would go all the way back to that point … It was a market-driven, activate the consumer type of model. You could argue that was an early version of personalisation’. Our interviews encouraged interview participants to explore when they first encountered policies or techniques that were labelled ‘personalized’, and the introduction of markets and market behaviours within government services was one measure used; associating diverse outcomes with values of choice and control for users.

Having left an NGO Richard worked for one of the world’s largest private sector consultancy companies. There he advised global companies invested in the ‘dot-com boom’ and learnt of their strategic plans ‘to really understand a consumer’s motivations at a really granular level … to try and help the company to target really narrow, to activate the consumer at the point where they're buying into it’. It was in this private sector context that he first encountered words like ‘personalized’ and ‘personalization’ and their close connection to technologies used to gather ‘granular’ consumer data to identify and predict moments when different market segments emerge; targeted marketing and advertising campaigns to stratified consumer groupings. He recalls industry colleagues being ‘very excited about the idea that you could aggregate data and target people at a market of one, at a personal level’. It was during this period that he got an ‘early entrée to the concept of targeting individuals … ’. When the dot-com bubble burst in 2001–2002 he moved into a temporary role in government, working on e-government projects that sought to enhance internet-based customer relations management systems in government services. He perceived an opportunity to combine improvements in IT, aggregate-level data analytics and service provision. Leadbeater, Mulgan and others were then advising the UK government but the policy environment was already sympathetic to the technological transformation of public service delivery, via improved communications and data analytics then used in the online marketing, sales and advertising industries. With others Richard established a private-sector consultancy, a bridge between industry and government, to establish a more aggregated view of people’s needs and a targeted set of services for UK citizens.

If one definition of personalization involves targeting the right intervention, to the right person, at the right time, Richard focused on avoiding costly and potentially harmful interventions, aimed at the wrong people at the wrong time: ‘overspending and underspending, inappropriate placements, and it leads to poorer outcomes’. He explained that every year approximately 30,000 children in England enter care and are looked after by local authorities. Using a statistically representative sample of cases, Richard’s organization showed local government officials that children have been placed in care needlessly. This analysis helps local authorities identify where they need more purposive data collection. One model replaces written notes about a person’s needs with a grid or ‘radar chart’ of metrics that account for education, mental health, vulnerability and other needs, which are numbered and ranked 0–10 in severity. These are used to build ‘holistic’ profiles for individual children that can be disaggregated and aggregated according to whether decisions are made on an individual or commissioning level. In this case, Richard explained, care providers are able to create more accurate and more preventative interventions, rather than rely on one-size-fits-all models.

Progress or decline for an individual are tracked iteratively and comparatively, over time. In principle, areas of acute need can be addressed before a crisis occurs. These metrics are viewed as a ‘breakthrough’ in the sector because they combined standardized metrics that are mobile across care settings and agencies, allowing the status of individuals to be tracked over time. These numbers help to improve communication and debate over so-called ‘intangibles’ which may be otherwise ignored. Richard can also take existing cases and establish radar chart metrics retrospectively, thereby testing the model against ‘real-world’ outcomes to improve predictive accuracy over thousands of cases in different authorities. It is possible to create risk prediction scores for emerging profiles; using cohorts to make predictive judgements about individual outcomes based on how individuals compare in terms of their holistic needs. The aim of this ‘personalized’ and data-driven approach to social care is prediction and prevention, based on emergent sets of comparative attributes. Each application of the model generates feedback for individuals and to the model overall, ‘every time you implement it, it gets another tweak, improvement, iteratively enhanced … We have more insight, more data to draw on.’ The person targeted and the interventions based on tracking their behaviour becomes measure via an evolving set of statistical comparisons and adjustments.

Richard’s interventions are implemented by local authorities, within the public sector and in reference with the financial and regulative duties of local government. They borrow and adapt ideas from the private sector, tracking and testing predictive measures of behaviour via n of one models. They do this using historic and existing datasets, enhancing and combining data within existing systems of service delivery. In terms of principles accompanying such practices, Richard highlighted how welfare systems aspired to a ‘one-size-fits-all’ form of universalism that also excluded many, misallocated resources and left others underserved as new kinds of demand emerged due to demographic and other trends. It became clear to Richard that state intervention requires a relational approach, that uses the individual desire to improve life and match this with specific services or support. What is highlighted by Richard’s story is how matching may involve a statistical achievement but the data required and the process of implementation within services and people’s lives are formed by historical relations between people, state and market.

*It’s a bit like going in Sainsbury’s with a shopping basket* – **Mary, hospital manager**.

Participants were not recruited to the study to form or confirm a consensus. Some wanted to explain that there were different interpretations and conflicting practices that have formed their careers, and it fell to them to balance competing priorities. For example, Mary first trained as a psychiatric nurse in the late 1970s and qualified in general nursing in the early 1980s. In terms of patient care, she explained, there was a long history in the health professions of ‘person-centered’ and ‘holistic’ care. These were sometimes (though not always) elided with ‘personalized’ care and they were attributed values throughout her professional life; respect, choice, shared decision-making and consent. ‘Personalization’, explained Mary ‘should be about holistic care’. Sensing emerging opportunities for experienced clinicians in management roles in the National Health Service (NHS), Mary left her nursing career and went to business school. This experience also affected her view of what personalization meant at different scales of person and system. Care was not only managed between people. Interagency coordination and multidisciplinary working ensures care ‘come[s] from a number of different quarters. Obviously, doctors could be important in that and nurses could be important, physiotherapists, occupational therapists, the voluntary sector, the family and the local community could be involved … ’ To manage those different stakeholders, a system of management, oversight and evaluation was required – care required financial co-ordination, evidence and data, research and resource management. Mary's reflections on management echoed Leadbeater’s vision for a public sector as a contained space, overseen by the state and served by a mix of providers rested on measures of efficiency. For Mary, too, ‘patient-centered’, ‘holistic’ and ‘personalized care’ reflected both an ethical relation between clinician and patient but were also associated with effective systems of bureaucracy that measured activities and allocated resources (‘personalized money for personalization’).

Mary explained that hospital managers employed techniques applied directly from industrial management to classify patients according to costs, to create financial incentives to increase their efficiency and improve quality. New valuation and accounting methods meant costs were attributed to hospital activity, laying the basis for financial data used to ‘track’ patients. First developed in the United States and called Diagnostic Related Groups (DRG) during the 1970s and 1980s (Fetter et al. 1980), a similar system was implemented in the early 1990s that the NHS called Healthcare Resource Groups (HRG) (see Grašič, Mason, and Street 2015). Patient care is costed according to financial categories – the cost of care and treatment patients receive, rather than the number or variety of specialist interactions required. These groupings draw equivalents between procedures and shape management abilities to predict different groups and groupings of patient according to need (HRGs are called a ‘prospective payment system’). As a financialised coding system HRGs became integral to audit and reporting standards between providers and the Department of Health. The consequences of their implementation for healthcare systems and individual providers have been substantial, contributing to operational and performance metrics and other rankings. Yet the underpinning logic of HRG comparisons is largely unknown to hospital patients and staff. They are value systems that shadows a system of pricing, while also contrasting with values of transparency and informed consent that are a common basis for ‘personalized care’ between clinician and patient.

DRGs and HRGs are an application of industrial management techniques that have tried to place quantifiable values on the ‘product’ of hospital performance: clinical care. Mary explained HRGs in terms of retail experiences, where diagnostic groupings led to valuing care based on different ‘baskets’ of activities like food in a supermarket shopping:

It’s a bit like going in Sainsbury’s [a large UK food retailer] with a shopping basket, you throw … “Right, a lemon and an orange and a banana are roughly the same size. They’re completely different, but actually they’ll do so I’ll stick them in the basket.” Then you say, “Actually a head of cauliflower and half a dozen potatoes, they’re more or less the same price so I’ll put those in the basket.” That’s my basket, that’s my diagnostic related group … Rather than going down and saying, “This is the actual cost of a lemon, this is the actual cost of a banana.” It bunched. The HRGs, and the DRGs before that, tried to bunch these similar types of diagnostic – hence they were diagnostic related groups or health related groups – into a kind of package. Therefore, then you could begin to measure what it was you were dealing with.

Collecting data on costs via HRGs allowed commissioners to track, monitor, compare and make efficiency savings within and between hospitals, based on ‘average’ people being treated in ‘average’ hospital trusts. In Mary’s experience as an NHS manager, this accounting system was fundamental to what became called personalization because it laid the groundwork for data-driven approaches to hospital management. The data systems were created to ask managerial and clinical questions: ‘Do you know who the people are that have got long term conditions, who have got a plethora of life-limiting conditions? How would you know where you should focus your personalised care?’ Providing holistic care and targeting care to those in need required a system of data collection, an infrastructure that was developed throughout the 1990s and 2000s for financial purposes to increase efficiency and equity between providers, that now remains central to NHS financial architecture for all models of care – personalized or not.

*They want to do it through apps, whereas this is primarily relational, you know?* – **Chris, NHS England policymaker**.

Like Mary, Chris was an NHS consultant who also used terms like ‘person-centred’, ‘tailored’ or ‘customized’ care to describe a clinical ethics based on mutuality and shared decision-making. Chris recognized that these terms had existed in the healthcare sector for a long time. They appeared to be synonyms but were also unreliable and confusing. People he worked with tend not to see a continuum of practice to connect them all: ‘I don’t think people out in the [NHS] system are using the phrase ‘personalized care’’ he said, ‘they're still saying “person-centred care”, or “personalization”’. He was a doctor for many years before working in health policy. He explained that NHS England’s policies on personalized care, such as the *Universal Personalized Care: Implementing the Comprehensive Model* (2019), brought together social and health policies under ‘personalized care’ after different government departments merged in 2016. The words, practices and histories involved do not overlap easily. He tended to use simple, non-policy language when working with other clinicians, policy makers and advisors, since the policy terms are ultimately ‘shortcuts’ for everyday practices, and practices have long and complex histories.

Chris’ professional history involved exploring, challenging and moving away from what he called ‘the medical model’ of rigid standards and evidence-based regulation of interventions. When supporting patients in the 1990s who were suffering from severe pain – ‘pain that medicine couldn’t explain’ – he was led to adopt more psychosocial approaches in his clinical practice. In his view, good clinical practice involved enabling people to develop independence, managing their health and integrating health psychology in ways that allowed people to live well with their conditions. Person-centred care was attitudinal as well as collaborative. It involved the ‘relational blurring between the medical and the psychosocial … taking actions that fit within a person’s values and preferences and context’. Person-centered clinical practice exposed tensions between standards of medical evidence, based on averages across populations, and establishing individual preferences and atypical thresholds of efficacy. Chris explained that medical education encouraged doctors to be ‘knowledge factories’ who could diagnose diseases and recommend drug A, B, or C, according to protocol. Chris said, ‘my job is to support you to consider those choices and then to tell me what you feel about them, and then we have a conversation about it’. He saw this model of personalized care as one that negotiated medical treatment and opened up non-medical choices, some of which may not be pharmaceutical.

Other portraits highlight constraints on choice that were financial, professional, regulatory and organizational and other interview participants noted gaps between rhetorical promise of some terms and their practice in the ‘real-world’. Chris admitted that practices and histories of personalized care based on meeting needs had ‘some really fuzzy edges’. Choice in medicine is constrained and regulated according to clinical evidence and resources. Access to some personalized care policies, such as direct payments and budgets, were rationed based on assessment criteria. NHS England’s *Universal Personalized Care* allowed some people more choice and control than others. In research-intensive healthcare systems such as the NHS, where novel therapies and other studies are introduced on a routine basis, different categories of patient develop rapidly, unevenly, or combine unexpectedly with others (see Day et al. 2021). Compounding these tensions Chris saw a distinction between the interpersonal ‘personalized care’ – guided by ‘fuzzy’ and relational work of establishing patient preference – and data-intensive ‘personalized medicine’ driven by what was glossed as ‘medical technology AI stuff’ that was being introduced by different groups within the Department of Health and Social Care, NHS Digital and NHSX.^5^ It was not only that there were ideological and ethical differences in terms of what being is defined as ‘personalized’ but differences in priorities, levels of funding, and legal responsibilities that are fragmented across different government agencies. The development and application of machine learning, Artificial Intelligence (AI) and other data-intensive practices within the UK health care sector were singled out as ‘dragging the system in that direction, frankly away from a more personalized, person-centred approach. And I think all we’re doing is rebalancing it, at the moment’. Emerging technologies used to measure and monitor health conditions computationally, tracking and automating decisions via large-scale datasets, signalled the opposite of an interpersonal, relational, preference-driven approach to clinical care. These were not only viewed as differences in how personalized care was being developed, or hindered, by competing practices, but these tensions also reflected limitations to bureaucratic powers that seek a unified, clinical culture for personalized healthcare and medicine.

*Where you are linking real GP data, A&E data and Facebook data, etc. The technology exists. It’s just a matter of can you connect all those things into a meaningful way* – **Susan, management consultant**.

Susan trained as a clinician but realized during medical school that she would not enjoy a career as a doctor. She looked for an alternative career soon after qualifying. Retraining as a management consultant, we met when Susan worked at one of the world’s largest management consultancies. There she specialized in advising public and private organizations in the healthcare sector, including the UK’s Department of Health and Social Care. Her definition of personalization was broad and the examples tended to be drawn from commercial companies and industries, outside the healthcare sector. Personalization involved ‘being offered something that is meaningful to you or that you require’; mobile phone technologies, online media platforms and retail recommendation systems all seek to satisfy this definition, often as quickly and efficiently as possible. Like Mary, she saw the ability to segment and track patients as a fundamental requirement to how value is understood in the NHS. Contemporary data systems are built upon previous systems of data collection, storage and analysis, leading to a complex patchwork of NHS data infrastructures that have served different purposes over time. In the early 2000s, Labour governments introduced further performance measures and targets to raise overall standards and improve healthcare equity, producing datasets related to provider operations and care quality. In the later 2000s performance measures were repurposed or enhanced with finer-grained forms of tracking. In her view, patient data across different care settings was becoming ‘much more customised, as it were, and patient sensitive … The first wave “let’s make sure failing hospitals or failing clinics get their act together and get them up to a certain bar”. Once that happens, how we now improve the service to make it more personal to an individual department’. This was a further illustration of how personalized approaches build on, leverage and develop previous systems of management.

In contrast to Chris, for whom patient choice was fundamental to person-centred care and who viewed data-intensive technologies as antithetical to choice, Susan argued that patient-centred care can be achieved if patient data can be linked and combined with other data types. For this to be a possibility, data from primary, secondary and tertiary NHS care contexts require consolidating. Data needed to be combined with social media and other commercial platforms. The use and reuse of data was key to increasing competition and providing NHS patients more choice in services. A different role was imagined for the state, which enabled data surveillance to be continuous and data to be interoperable, such that a government’s ideal role is one of infrastructure provider, ensuring common system standards for third party organizations to access a shared platform. Legacy systems with no future compatibility should be discarded as soon as possible. Government investments in a data-led healthcare system should therefore focus on data infrastructures that underpin all systems. This was expected to generate greater predictive interventions through linking data, with diagnostics becoming an in silico achievement. Another definition of personalized care, as a technology, develops out of this digital context: ‘can you connect all those things into a meaningful way and then predict somebody’s mental health or condition before it arises?’. The large-scale harmonisztion of routine health, research, novel behavioural and social media data would accelerate personalized, predictive and preventative health systems. Susan explained that once data had been harmonized in this way then it was possible to start doing ‘very, very powerful analytics’:

If you have this type of profile, this type of genome, this type of ethnicity and you have these parameters in your GP records, it will suggest that you are going to become (a) depressed or (b) obese. Whatever it may be, you can see that playing out, both from the organic side as well as the behavioural side.

Then, if you integrate Facebook and those types of social media, you can get a very, very predictive algorithm of what life you’re likely to have. Facebook can predict depression earlier now than a doctor can predict that you’re likely to get this. Even though it’s unusual, that is possible. So I think those are the things that are likely to be more targeted in the preventative side.

Data linkage is a foundation for predictive analytics. In the interests of greater personalization at scale, Susan explained, NHS health and social data should be released to private industry. It was less clear, or by what basis, commercial data from social media platforms would be freely available to other data processors. It was taken for granted that effective levels of personalized prevention would be supplied by commercially developed apps and other software, with the UK government, the NHS and other public organizations acting as platforms for data-driven private sector innovation.

*You can provide the tailored product for a mass, which is something totally new* – **Alex, software entrepreneur**.

Like other portraits in this gallery, Alex had experience of working for one of world’s largest management consultancies. When he was interviewed for this study he led a technology company for managing adult social care, a communications and coaching app. After completing graduate studies at a prestigious US university he was attracted to the competitive challenge of management consultancy and consulted within a range of sectors. He desired what he called a more ‘socially responsible’ form of entrepreneurship, more closely related to previous roles at climate and conservation charities and voluntary organizations. He also drew on personal experiences of caring for elderly relatives and noted the difficulty of caring for older people in their own homes. He combined his professional and personal experiences with observed social trends about an aging society, political inaction in the social care sector, funding limits and growing constraints on the care workforce. So Alex foresaw a growing ‘social care crisis’ to be addressed by the commercial sector. He co-founded a software company seeks to manage the health and social care of elderly people in private care settings.

The majority of UK adult social care settings are operated by private companies. Their datasets are proprietary and tend not to be interoperable with other private or public sector datasets, except for statutory governance and regulatory purposes. Alex’s tech start-up was creating an app designed to serve as a reporting and communication device, shared between families, carers and older people. The testing phases of product development focussed on the care home context, where existing levels of trust are high in an already regulated context. Data quality was higher than if the product was launched direct to consumers, he said. While small numbers of people were using the app at the time of our interview, these users produced large quantities of data. The app also digitalizes and automates existing, routinely collected data as part of care management and required by care home regulators. But the app extends beyond current standards of data collection to track individuals and provide alerts based on population, cohort and individual level parameters that change as users update their app profiles. For instance, data is collected on sleep patterns, nutrition and hydration and toilet use; these data can be used to create alerts for risks such as urinary infection. Alerts for individual users are triggered by these dynamic data according to prior patterns that aggregate singular and multiple user data, whose performance improves with further updates from all users. Advanced notifications were called a kind of monitoring or ‘coaching’ – scarcely different to ‘nudging’ behavioural change based on cohorts and other population datasets. The aim is for millions to use this app while being cared for at home, where currently there is little reliable data to inform remote care access. Since the company was being financially supported by a large health insurance company, the data could be used for a range of different and connected purposes: to further develop the app’s accuracy in preventing health emergencies via app notifications; to ‘coach’ individuals into improved health behaviours; to penalize or reward policy holders with adjustments to their insurance premiums.

An elderly person’s home was characterized as being data scarce relative to other domains of care. This was presented by Alex as an opportunity and as a challenge to the development of a ‘personalized’ care app: ‘assess[ing] what’s going on in the house and that enables us to detect patterns of behaviour’. He explained how the company navigated difficult ethical and technical terrain – justifying the introduction of potentially intrusive data surveillance technologies via the app, while also proving the value of preventative notifications to users who may yet feel fit and healthy. Alex saw potential throughout the app to learn and therefore intervene in the lives of app users. This was a question of applied data science: ‘I’m just talking about looking at the data properly and having a data scientist, project manager, product engineers and two geriatricians in the room thinking, “What are the issues that arise most often in the house of the older adults, and how can I anticipate? What are the symptoms? Can I see the symptoms, the data that I’ve got, and if yes, let’s push and learn and address it”’. At this early phase of development, the app was a research tool – aimed at understanding the extent to which preventative, ‘personalized’ health interventions could be routinely automated. Overall, collecting large amounts of very detailed data provides an enormous expansion in potential uses of the app. The company’s data becomes an asset to be prospected, reused, repurposed: ‘That’s the kind of data I want to use now’, explained Alex, ‘plenty of potential for later on, but that’s the data I want to use. That’s the personalization I want to work on’. What can be personalized is entwined with the data of elderly life, a promise that depends on repurposing data in multiple contexts.

The benefits of the app to an individual user or group of users (i.e. family and other careers) extend via the assetisation of data into insurance markets. Predicting health outcomes, he says, is a big problem for insurers because the health of older people can be unpredictable compared to customers in their 30s. Personalization has been used to stratify pricing, as it has been in other commercial sectors. Insurers want more data for more flexible premium pricing into older age. When asked if the app could benefit insurers and allow them to increase prices for vulnerable adults he argued that preventing illness or long-term frailty would safeguard individuals. Showing users how their sleep is declining or how their exercise routines are helping them lose weight will benefit their heath management, which could even be linked to lower premiums: ‘The more data you share, the cheaper is your scheme’. While this might result in benefits to individuals or groups, a more preventative approach to adult social care could have significant impacts on public services, ‘We’re talking about massive savings for the NHS and social security service’.

The app was described as a ‘holistic platform’ that participated in a societal shift towards ‘mass personalization’ or ‘mass tailoring’. He noted streaming services like Spotify as a model, which provides a personalized service at scale. This was viewed as historically unprecedented, since the cost of high levels of customization usually resulted in higher cost products affordable by a relatively low number of people: ‘For the first time, you can have a personalized service or product for the mass thanks to technology which is brand new’. Across sectors, he argued, this shift was achieved via a steady fall in marginal costs towards zero, so that pricing access would not be affected by the cost of technology. It’s this market-driven innovation process that allows a steady shift from one-size-fits-all to generic or ‘mass personalized’ products, where the personalizing product is accessible to all yet differently experienced by everyone.

## 4. Discussion

How ‘personalization’ became associated with making government services ‘tailored’, ‘targeted’ and more ‘adaptive’ has been driven by diverse histories, many connected to hopes for fairer, more inclusive, efficient and less wasteful public services, services that enable greater choice and control for citizens. For our interview participants, personalization was not a straightforward synonym for privatization; the word ‘personalization’ was accepted by interview participants on specific terms, according to specific practices, some more or less cognate with others, and in ways that depended on the experiences of those we interviewed and their professional backgrounds. Comparing accounts of personalization has been useful when judging the importance of data-intensive commercial services and their influence on more ‘personalized’ public sector services. That influence was bidirectional, insofar as public sector services were formed on a one-size-fits-all basis, whose universal mandate and data standards laid the basis for innovations traditionally associated with the private sector. Though the impact of internet technology businesses were frequently discussed during our interviews, their influence on government was viewed as a distinctive yet inconsistent feature of late twentieth and twenty-first century bureaucracies, as administrations seek adaptive, flexible and ‘smart’ outcomes.

Such was the diversity in opinion that, even within the health and social care sector, it has been difficult to attribute a single meaning or set of practices to ‘personalization’. This is one reason why this article does not strive towards a general analytic or theory, since it has been fruitful to learn and confront this variety, its contradictions and uncertainties. These have long featured, as Leadbeater and Mulgan claimed in the early 1990s: ‘retailers have the technology to record thousands of real time changes in people’s preferences’ (1994, 61). Aside from the question of how, if or should governments adopt similar practices, Leadbeater had previously made ‘preference’ key to an ideology of democratic participation. By also using ‘individuals’ as the ‘progressive’ unit by which preference was attributed, he ensured that consumer behaviour and political action could share common performance criteria. It is not so much that these views led directly to attempts to ‘personalize’ public services but they are important assumptions that prefigured the technological possibility of doing so. Interviews with Richard, Mary, Chris, Susan and Alex highlighted how enduring professional codes, interpersonal ethics, and existing regulative and organizational frameworks found friction with both the commercial and public services they experienced. Their ability to apply data-intensive methods is part of this story of personalization, where other profiling techniques were reported in specific contexts, shaped by legal, regulatory and financial constraints. This was one benefit of the interview method used in this study. Another was that by asking interview participants to encode their views into a biography of work and practice, we were able to document the importance of moving in, across and out of the public sector to a more general history of personalization in the UK. It showed perspectives on personalization were shaped by changing settlements between public and private sector activity, particularly in terms of how data are created and connected.

The evolution of New Public Management (NPM) in the UK is one context that provides a chronological ground for many of our interviews, and its parallel history can help to make sense of the contrasting and contradictory views related to what personalization should involve. First identified in the 1980s and codified in the 1990s, NPM names a series of trends that affected western economies during the late twentieth century: a reduction in government growth in terms of public spending and staffing; a shift toward privatization or quasi-privatization with renewed emphasis on decentralization in service provision; the development of automation, particularly in information technology and in the distribution of public services; and the development of an international approach to public management, policy design, decision styles and inter-governmental cooperation (see Ferlie 2017; Hood 1991). These ‘megatrends’ in government were said to define NPM with the ‘attempt to implement management ideas from business and private sector into the public services’ (Lapuente and Van de Walle 2020). This has transformed what governments do, who governs and how – shifting relations between states and citizens, public and private industry.

The rapid introduction of NPM during the late 1980s and 1990s followed a period of vocal opposition by politicians and business leaders to the management of the public sector. When supermarket director Roy Griffiths reviewed NHS provision in 1983 he complained about its management structure: ‘if Florence Nightingale was carrying her lamp through the corridors of the NHS today, she would almost certainly be searching for the people in charge’ (Griffiths 1983, 12). NHS hospitals, went the argument, should be run like supermarkets or other large commercial organizations, preferably by managers with experience of private sector techniques of improving efficiency, quality and safety. Mary became a hospital manager at the same time Leadbeater was writing in *Marxism Today*, and experienced the implementation of industrial management ideas in NHS hospitals first hand – grouping patients according to a shopping basket costing method. More or less simultaneously, Griffith’s review of NHS provision (1983) and social care (1988) were translated into policy via the Conservative government’s white paper *Working for Patients* (1989) and then implemented as the National Health Service and Community Care Act (1990). These gave a substantial role to NPM in transforming public and private sector healthcare in the UK. Legislation brought important changes: the separation of organizations that commission and those that provide services (known as the purchase-provider split or ‘internal market’); local delegation and self-governance of hospitals as independent hospital trusts; permission granted to hospitals to earn revenue from services; independence in the rates of staff pay; allowing money to ‘follow the patient’ across administrative boundaries that required accounting systems to track patient activity in multiple settings; large GP practices could block purchase services from hospitals; rigorous audits of service quality and value for money would mean government would be intervene on matters of quality (Department of Health 1989; Health Foundation 2021). Susan explained that NPM performance metrics formed the basis for contemporary NHS infrastructures and stressed the need to further centralize different data types across settings as a prerequisite for a more data-intensive and personalized healthcare system.

The policy architecture of ‘personalized public services’ took many years – the National Health Service and Community Care Act (1990) introduced quasi-markets to stimulate private competition in the hope that this would provide service users greater choice and control (see Hudson 1992; Le Grand and Bartlett 1993). Richard’s relationship to personalization is partly formed by trends central to this history of public sector management – highlighting ways that public sector reform and technological change have co-evolved. NPM partly explains Richard’s ability to move between sectors, apply commercial techniques in public sector services, and produce data that is valued by local authorities because local authority services are now split into multiple quasi-markets. Alongside the data practices that Richard employs, structural changes have affected how social workers assess an individual’s circumstances in order to determine what services are appropriate to their needs, rather than fitting a whole individual to a single service. Throughout the 1990s, data about service users also became collected to develop a personal profile of needs (Barnes 1998; Rummery 2002), driven by incentives of public sector audit, standardization and performance measurement (Clarke, Gewirtz, McLaughlin 2000). From this point of view, the n of 1 radar-dial of metrics used to track user needs forms a response to how NPM transformed one-size-fits all, service-led decision-making. It supports a ‘needs-based’ approach to assessment that is the legacy of NPM (Barnes 1998; Middleton 1997). Computational techniques used to form recommendation systems contribute to and are implemented within this broader approach to citizens. In this sense, NPM acted to stimulate the legislative, corporate and technological division of needs. It did so by formatting citizens within a divided market.

Many of our interview participants noted material shortfalls in the provision of personalized services – budget constraints, a lack of co-ordination and transparency between organizations, fragmented performance standards, a lack of data and data interoperability. Accusations of service-level fragmentation are also frequently levelled at NPM in general. Negative evaluations of new management techniques leads some public administration scholars to declare a post-NPM era (see Christensen and Lægreid 2007), whereby solutions are sought to correct NPM’s contradictions. The timing of our interviews (2018–2020) and the cohort of individuals we interviewed meant interviewees tended to be familiar with the steady yet uneven marketization of public sector services 1990–2020. The failures and successes they attributed to public sector personalization were entangled in the legacies of NPM. This meant that common criticisms of NPM – multiplied providers (‘agentification’) and separations between public finance provision; competing types of public, private, charitable care production (‘disaggregation’) – are a consequence of creating specialist sub-markets that sought to target services to users.

Though they differed in how they defined personalized care, Mary, Chris and Susan complained that it was being thwarted by contradictory actions of different providers or agencies. Countering these negative trends has involved improving coordination vertically between government and other actors horizontally. This has been described as characteristic of post-NPM approaches, which seek greater inter-agency coordination and a holistic management style, fostering boundary-spanning skills and joined-up targets for ‘whole of government’ or ‘joined-up government’. These measures aim to improve a steering capacity at the centre of government, while civil servants are imagined as network managers and partnership leaders, rather than seeking to enact a pure business manager role sought under the NPM model (see Christensen and Lægreid 2007; Lodge and Gill 2011). The portraits all touch on how the barriers to personalization – competition between providers, the fragmentation of care, data systems and IT procurement – are legacies of NPM as much as they are legacies of traditional welfarism as such. Data interoperability is presented as one but not the only solution to these barriers. Personalization endures in this post-NPM landscape, as both tradition and ambition, as do management consultants in this and many other areas of public sector life.

One significant consequence of post-NPM reforms has been the emergence and adoption of digital-era government, loosely articulated by Susan’s recommendations to centralize, privatize, and combine all data, and exemplified from a provider’s point of view by Alex. Digital-era government imagines the state as a platform that providers and citizen users plug into – a data infrastructure, an in silico space of competition and innovation, that allows data to pass through an operational centre. The UK Government Digital Service (GDS) was established in 2013 with reference to Tim O’Reilly ‘lessons’ for what he called ‘Government as a Platform’ (2011). As a technology incubator and regulator, ‘a Government 2.0 approach would use open government data to enable innovative private sector participants to improve their products and services’ (2011, 34). GDS declares itself to be building ‘platforms, products and services that help deliver a simple, joined-up and personalized experience of government to everyone’ (UK Government 2022). The focus of government should be opening up data sources and promoting a common, core infrastructure of shared and interoperable digital systems, technology and processes; a platform ‘on which it’s easy to build brilliant, user-centric government services’, as one former GDS director argued (Bracken 2015). These government services are imagined to be unencumbered by prior investments in older or established material infrastructures. They are not contained or inhibited by physical parklands, nor are they burdened by the historical legacies of previous attempts to deliver a ‘simple, joined-up and personalized experience of government’. Matters of efficiency are not a posthoc judgement made by an executive class of government employees. This is a government that forms in a digital cloud to enable private innovation in ‘the equivalent of a thriving bazaar’ (O’Reilly 2011), where participants can be both producers and consumers, and where ‘personalization’ is rendered a process rather than a determined outcome.

While rhetorical novelty prevails, amidst innovation-driven approaches to government, our interview participants reflected on relations between ‘one-size-fits-all’ services and more recent data-driven platforms. Rachel Faulkner-Gurnstein and David Wyatt (2021) have described how the ‘platformisation’ of UK health services involves ‘numerous aspects of the health service – its facilities, its reputation, its relationship to patients, and perhaps most prominently, the data that it produces – are transformed into assets and entered, in diverse and complex ways, into newly constituted economic circuits’. Legacy aspects of past healthcare services, perhaps most prominently the data formed via NPM parklife managerialism, remain in these economic circuits whether or not personalization is the aim.

## 5. Conclusion

I began this article with a picture of a state whose park-like enclosure allowed government authorities to manage a closed, managed-system of private enterprise. Initially, Charles Leadbeater did not specify the technology required for his everyday urban utopia. Some years later he would champion technologies that could collect, analyze and rapidly translate behavioural data into more responsive and adaptive services. He and his colleagues would call this transition ‘personalization’. By interviewing professionals from various health and social care contexts, I have been tried to map a small sample of languages, practices, histories and technologies associated with this term, some rather old and familiar, others still held in promissory futures.

Leadbeater’s writings described an historical transition from NPM to mass personalization. These portraits reflect the unevenness of that transition. It has also shown how NPM helped to standardize, personalize, privatize and also fragment different parts of government provision, meaning that the goal of ‘tailoring’ services using market discipline also divided the needs of its beneficiaries. Writing in 1988 or 2004 it was not easy for Leadbeater to foresee mass participation via the emergence of government as platform. Investments in a more ‘personalized’ state, whether as parklife government or platform government, survive despite such changed relations between state and market.

It is not straightforward how, in the words of Antoinette Rouvroy and Thomas Berns, ‘under cover of ‘personalizing’ information, service and product offers, in the algorithmic governmentality era one is rather witnessing a colonization of public space by a hypertrophied private sphere’ (Rouvray and Berns 2013, 165). At least from the vantage point provided by these portraits, the historical era of algorithmic public administration has not been fully formed in the UK. Personalizing information must also be embedded in a variety of practices, amidst the lives of workers whose sense of an era may include many different historical pasts and possible futures.

These interviews did not find evidence of personalized services based on a template provided by Silicon Valley companies. They did not define personalization as being exclusively or necessarily algorithmic in delivery, although some certainly envisioned a role for computational, automated or semi-automated personalized services. The desire for personalized outcomes were narrated according to many material, ideological and administrative histories, prior to and via their potential realization in information technology. No doubt the biographical method used in this article has allowed me to stress how people’s values, their professional lives and practices are key to understanding how personalization is both witnessed, and how it traverses (and contorts what we may conceive as) ‘spheres’ of public and private life. As a consequence, the history of personalization presented by this article has engaged in the historical, ethical and political connections with, and long-established desires for, a less wasteful, more tailored, inclusive and responsive form of service provision. It has been in these mixed, partial, shifting and contested histories that ‘personalization’ has helped to reshape life in the UK in the last 40 years.

## Notes

1 *Marxism Today* was founded in 1957 by the Communist Party of Great Britain. It ceased publication in 1991. Among other achievements, it cultivated British cultural studies and published work by Stuart Hall, Eric Hobsbawm, Beatrix Campbell and others. In 1988, its monthly circulation was approximately 20,000 copies. *Marxism Today* is credited with introducing the term ‘Thatcherism’ to describe a combination of fiscal, monetary, industrial and cultural trends that influenced British political life in the 1980s and 1990s (see Harris 2015).

2 Leadbeater advised Peter Mandelson when the Labour Party first adopted the campaign slogan, ‘For the Many Not the Few’ featured in the 1997 Labour Party manifesto and later revived as part of Jeremy Corbyn’s 2017 and 2019 election campaigns.

3 The interview study ‘What is Personalisation?’ had ethics approval granted by The Research Ethics & Integrity Sub-Committee (REISC) at Goldsmiths, University of London (Reference 1484).

4 Every effort has been used to preserve the anonymity of study participants. All names are pseudonyms.

5 NHSX established early 2019 at the time of interviews.
